# A Serum Multi-Parametric Analysis Identifies an Early Innate Immune Signature Associated to Increased Vaccine-Specific Antibody Production and Seroconversion in Simultaneous COVID-19 mRNA and Cell-Based Quadrivalent Influenza Vaccination

**DOI:** 10.3390/vaccines12091050

**Published:** 2024-09-13

**Authors:** Martina Severa, Daniela Ricci, Marilena Paola Etna, Marzia Facchini, Simona Puzelli, Giorgio Fedele, Egidio Iorio, Giada Cairo, Sara Castrechini, Valentina Ungari, Marco Iannetta, Pasqualina Leone, Mattea Chirico, Maria Elena Pisanu, Barbara Bottazzi, Livia Benedetti, Michela Sali, Remo Bartolomucci, Stefano Balducci, Cecilia Garlanda, Paola Stefanelli, Antonietta Spadea, Anna Teresa Palamara, Eliana Marina Coccia

**Affiliations:** 1 Department of Infectious Diseases, Istituto Superiore di Sanità, 00161 Rome, Italy; daniela.ricci@iss.it (D.R.); marilenapaola.etna@iss.it (M.P.E.); marzia.facchini@iss.it (M.F.); simona.puzelli@iss.it (S.P.); giorgio.fedele@iss.it (G.F.); giada.cairo@iss.it (G.C.); pasqualina.leone@iss.it (P.L.); paola.stefanelli@iss.it (P.S.); annateresa.palamara@iss.it (A.T.P.); 2 High Resolution NMR Unit, Core Facilities, Istituto Superiore di Sanità, 00161 Rome, Italy; egidio.iorio@iss.it (E.I.); mattea.chirico@iss.it (M.C.); mariaelena.pisanu@iss.it (M.E.P.); 3 ASL ROMA 1, Regione Lazio, 00145 Rome, Italy; sara.castrechini@aslroma1.it (S.C.); valentina.ungari@aslroma1.it (V.U.); studiobartolomucci@gmail.com (R.B.); antonietta.spadea@aslroma1.it (A.S.); 4 Infectious Disease Clinic, Tor Vergata University Hospital, 00133 Rome, Italy; marco.iannetta@uniroma2.it (M.I.); livia.benedetti27@gmail.com (L.B.); 5 Department of Inflammation and Immunology, Humanitas Clinical and Research Centre—IRCCS, 20019 Milan, Italy; barbara.bottazzi@humanitasresearch.it (B.B.); cecilia.garlanda@humanitasresearch.it (C.G.); 6 Department of Laboratory and Infectious Sciences, Fondazione Policlinico Universitario A. Gemelli IRCCS, 00168 Rome, Italy; michela.sali@unicatt.it; 7 Department of Basic Biotechnological Sciences, Intensive and Perioperative Clinics, Università Cattolica del Sacro Cuore, 00168 Rome, Italy; 8 Metabolic Fitness Association, Monterotondo, 00015 Rome, Italy; s.balducci@hctdiabete.it; 9 Department of Biomedical Sciences, Humanitas University, 20090 Milan, Italy

**Keywords:** COVID-19, influenza virus, vaccination, innate immunity, metabolism

## Abstract

In this pilot study, a multi-parametric analysis comparing immune responses in sera of adult healthy subjects (HS) or people with type 2 diabetes mellitus (T2D) undergoing the single or simultaneous administration of mRNA-based COVID-19 and cellular quadrivalent inactivated influenza vaccines was conducted. While SARS-CoV-2 antibodies remains comparable, influenza antibody titers and seroconversion were significantly higher upon simultaneous vaccination. Magnitude of anti-influenza humoral response closely correlated with an early innate immune signature, previously described for the COVID-19 vaccine, composed of IL-15, IL-6, TNF-α, IFN-γ, CXCL-10 and here extended also to acute-phase protein Pentraxin 3. People with T2D receiving simultaneous vaccination showed a protective response comparable to HS correlating with the early induction of IFN-γ/CXCL10 and a significant reduction of the circulating glucose level due to increased oxidation of glucose digestion and consumption. These data, although preliminary and in-need of validation in larger cohorts, might be exploited to optimize future vaccination in people with chronic disorders, including diabetes.

## 1. Introduction

Due to the highly likely re-occurrence, in the next years, of COVID-19 seasonal infection, as observed for other common respiratory viruses [1], the implementation of COVID-19 vaccination programs in European countries, with the use of updated vaccine compositions based on predominating SARS-CoV-2 lineages, has broad clinical and economic benefits and will enable medical preparedness and targeted public health strategies to mitigate COVID-19 transmission.

Analogously, seasonal influenza (flu) vaccination is considered one of the most effective means of reducing the burden of disease, which causes an estimated 4–50 million known symptomatic infections in the European Union/European Economic Area each year, and 15,000–70,000 deaths of European citizens from flu-related causes [2].

Starting from the 2021–2022 northern hemisphere flu season, more than 20 European countries adhered to the European Centre for Disease Prevention and Control (ECDC)/European Medicines Agency (EMA) joint statement and indication on the possibility to simultaneously administer vaccinations for COVID-19 and the flu (extended during the autumn 2023 vaccination campaigns also to the currently authorized COVID-19 vaccine composition for new SARS-CoV-2 variants, EMA/257222/2023).

So far, different cohort studies have assessed the safety and immunogenicity of the concomitant administration of COVID-19 vaccines (protein-, adenoviral or mRNA-based, i.e., NVX-CoV2373, ChAdOx1, mRNA-1273 or BNT162b2) and seasonal flu vaccines in adults or older people receiving the quadrivalent cell-based flu vaccine (Flucelvax) or the adjuvanted trivalent flu vaccine (Fluad), respectively [3,4,5,6,7,8]. No safety concerns or immune interference were found for any of the vaccine combinations or ages of the vaccinated subjects (65− or 65+).

From an immunological point of view, the simultaneous administration of heterologous vaccines may result in potentiating vaccine-specific immune responses in several ways: by stimulating innate cell compartment and bystander responses via the release of inflammatory cytokines and by the activation of trained immunity mechanisms that could enhance non-antigen-specific responses influencing metabolic, immunological and epigenetic changes [9,10].

Systems biology and multi-omics studies have identified molecular networks linked to early innate immunity that orchestrate protective immune memory in response to vaccination. These analyses conducted upon either COVID-19 or flu vaccinations are all concordant with the fact that early signatures induced within a few days after immunization predict and yield insights into the nature of vaccine-specific protective responses [11,12,13,14,15,16,17], but this aspect has been less studied in the presence of chronic disease.

In this pilot study, we conducted a serological multi-parametric analysis on healthy or type 2 diabetes (T2D) adult individuals undergoing the simultaneous administration of COVID-19 mRNA-based and cell-based quadrivalent flu vaccines. Here, the vaccine-specific humoral responses were compared to the early induction of inflammatory cytokines and chemokines, previously found to be regulated by COVID-19 vaccination. We also integrated these parameters with the analysis of circulating levels of the acute-phase protein Pentraxin 3 (PTX3) and of metabolites involved in glycolytic, liver, amino acid, as well as lipid and fatty acid metabolic pathways. In this study, we sought to assess whether an innate immune module could act as an early indicator of antibody response to single or simultaneous vaccine administration.

## 2. Material and Methods

### 2.1. Subjects’ Enrollment

All the subjects in the groups (*n* = 67) described below were adults ≤ 65 years old, with no autoimmune disorder or cancer and with no history of immunosuppressant/cortisoid treatment for at least the previous six months from first blood withdrawal. This multicentric study enrolled participants at four different sites in the Regional Health Area of Lazio, in Italy: Infectious Disease Clinic of Tor Vergata University Hospital (Rome, Italy), Fondazione Policlinico Universitario “A. Gemelli”, IRCCS (Rome, Italy), ASL ROMA 1 (Rome, Italy), Metabolic Fitness Association (Monterotondo, Rome, Italy). The demographic, clinical and lifestyle characteristics of the enrolled immunized subjects were collected at each visit and are listed in Table 1. T2D subjects were enrolled among those individuals with non-insulin-dependent type 2 diabetes mellitus that had been diagnosed at least five years previously, with glycated hemoglobin ≤ 7 and a body mass index of 27–40. Approval for the collection and study of blood from healthy and T2D vaccine recipients was obtained by the Istituto Superiore di Sanità Review Board (AOO-ISS-22/03/2021–0010978 and amendment AOO-ISS-19/03/2021–0010819). All the participants gave written informed consent.

The Flu*_before_*CoV group comprised of healthy subjects (HS) [*n* = 18; mean age ± SD: 52 ± 11; female/male ratio: 11F/7M (1.57)] to whom Flucelvax (season 2021–2022) was administered 1 month before immunization with the third booster dose of anti-COVID-19 BNT162b2 vaccine. The Flu*_plus_*CoV groups consisted of both HS [*n* = 19; mean age ± SD: 57 ± 8; female/male ratio: 11F/8M (1.38)] and people with T2D [*n* = 6; mean age ± SD: 59 ± 5; female/male ratio: 2F/4M (0.5)], to whom Flucelvax (season 2021–2022) was simultaneously inoculated in different limbs with the third booster dose of anti-COVID-19 BNT162b2 vaccine. CoV*_only_* group was composed of HS [*n* = 24; mean age ± SD: 44 ± 11; female/male ratio: 16F/8M (2)] to whom the only third booster dose of anti-COVID-19 Comirnaty vaccine was administered.

The flu vaccines administered were in accordance with WHO recommendations for the 2021–2022 northern hemisphere influenza season: a quadrivalent formulation containing the following viral strain: A/Wisconsin/588/2019 (H1N1) pdm09 virus, A/Cambodia/e0826360/2020 (H3N2) virus; B/Washington/02/2019 virus (B/Victoria lineage); B/Phuket/3073/2013 virus (B/Yamagata lineage) [18].

### 2.2. Serum Collection and Storage

**Figure 1:**
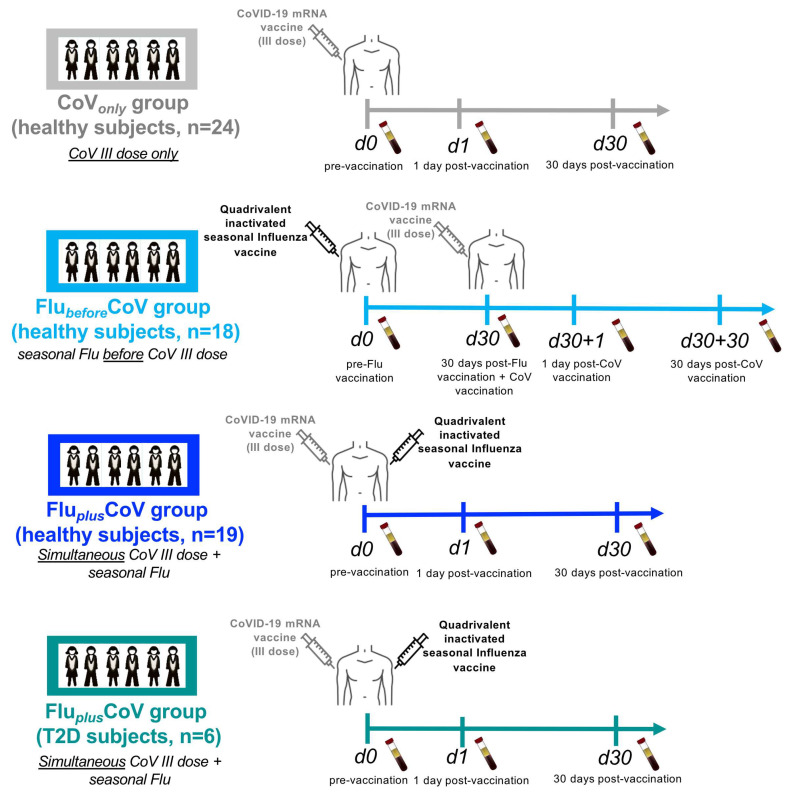
Scheme of enrollment and collected samples from healthy and type 2 diabetes mellitus (T2D) vaccine recipients. A schematic representation of groups, number of study participants and timeline of sample collection are depicted. “CoV*_only_*” group (in grey) comprised of healthy subjects (HS) (*n* = 24) to whom only the third booster dose of anti-COVID-19 BNT162b2 mRNA vaccine was administered. “Flu*_before_*CoV” group comprised of HS (*n* = 18) (in light blue) to whom the 2021–2022 seasonal cell-based quadrivalent influenza (Flu) vaccine (Flucelvax) was administered 1 month before immunization with the third booster dose of anti-COVID-19 BNT162b2 mRNA vaccine. “Flu*_plus_*CoV” groups comprised of HS (*n* = 19) (in blue) and of T2D (*n* = 6) (in petrol green), to whom 2021–2022 seasonal cell-based quadrivalent flu vaccine (Flucelvax) was simultaneously inoculated in different limbs with the third booster dose of anti-COVID-19 BNT162b2 mRNA vaccine. Demographic and clinical information as well as serum samples from longitudinal peripheral blood withdrawals were collected at the following time points: immediately before (day 0, d0), as well as 1 day and 30 days (d1 and d30, respectively) after the third booster dose of COVID-19 mRNA vaccine in the presence or absence of the flu vaccine. Only for “Flu*_before_*CoV” group sera were stored immediately before (day 0, d0) quadrivalent flu vaccine, as well as before (day 30, d30) and 1 and 30 days after (d30 + 1 and d30 + 30, respectively) the third booster dose of the COVID-19 mRNA vaccine.

Peripheral blood of enrolled HS and T2D was longitudinally collected in fasten conditions in serum separator tubes (BD Diagnostic Systems, Franklin Lakes, NJ, USA) before and after vaccination at the time points specified in Figure 1. Sera were obtained by centrifugation at 1800× *g* at 4 °C, aliquoted and cryopreserved at −80 °C for future analyses.

### 2.3. SARS-CoV-2 IgG Immunoassay

Serum samples collected at various intervals before and after vaccination/s were tested using the DiaSorin Liaison SARS-CoV-2 trimeric Spike (S) IgG assay (DiaSorin, Saluggia, VC, Italy). This two-step chemiluminescence immunoassay (CLIA) is designed to detect IgG antibodies targeting the native trimeric form of the SARS-CoV-2 S protein. Testing was conducted using the LIAISON^®^ XL analyzer, which measures the concentration of SARS-CoV-2 trimeric S IgG in binding antibody units (BAU/mL) [19]. The maximum value in the assay range is 2080 BAU/mL. Values ≥ 33.8 BAU/mL were interpreted as positive according to the manufacturer’s instructions. If results exceeded the assay range, the samples were automatically diluted 1/20 and the test was repeated.

### 2.4. Hemagglutination Inhibition (HAI) Assay

Specific anti-flu antibodies against all the vaccine antigen components, included in the Flucelvax Quadrivalent influenza vaccine used in the 2021–2022 season [A/Wisconsin/588/2019 (A/H1N1pdm09), A/Cambodia/e0826360/2020 (A/H3N2), B/Washington/02/2019 (B/Victoria lineage) and B/Phuket/3073/2013 (B/Yamagata lineage)], were measured using a HAI assay, according to WHO standard procedures [20]. In brief, to remove non-specific inhibitors, the sera samples were treated with receptor-destroying enzymes (RDEs) (Denka Seiken, Tokio, Japan), incubated overnight at 37 °C and then incubated at 56 °C for 30 min to inactivate the RDEs. Two-fold serial dilutions of each RDE-treated serum sample, starting from 1:10 dilution, were tested for their ability to inhibit the agglutination of 0.5% turkey red blood cells (RBCs) by four hemagglutinating units of each egg-derived antigen. All tests were performed in duplicate and the serum antibody titers were recorded as the highest serum dilutions at which complete inhibition was achieved.

The flu vaccine immunogenicity [21,22] was analyzed as follows: (1) seroconversion rate as the percentage of vaccine recipients with either a pre-vaccination HAI titer < 1:10 and post-vaccination HAI titer ≥ 1:40 or a pre-vaccination HAI titer ≥ 1:10 and ≥ fourfold increase in post-vaccination HAI antibody titer; and (2) seroprotection rate as the percentage of vaccine recipients with a post-vaccination HAI titer of at least 1:40.

### 2.5. Serum Cytokine and Chemokine Quantification

The quantitative determination of cytokines and chemokines was conducted on stored serum samples from vaccinated HS and T2D by custom multiplex next generation ELISA by ELLA system (Bio-techne) [11], according to the manufacturer’s instructions. In particular, IL-15, IL-6, TNF-α, IFN-γ and CXCL10 were measured.

### 2.6. PTX3 Quantification

PTX3 circulating levels were measured, as previously described [23], using a sandwich ELISA developed in-house by personnel who were blinded to patient characteristics. The assay has a lower limit of detection of 0.1 ng/mL, inter-assay variability between 8 and 10% and it does not cross-react with CRP.

### 2.7. NMR Metabolomics Analysis

NMR-based metabolomics was performed on intact serum samples (diluted to 0.7 mL with 0.9% NaCl in D2O) and analyzed on a Bruker AVANCE NEO 600 spectrometer (14.1T; Bruker BioSpin, Karlsruhe, Germany) [24]. Three molecular windows approaches were adopted for each serum sample using: (a) a standard nuclear Overhauser effect spectroscopy pulse sequence NOESY 1D presat for both signals of low and high molecular weight molecules, (b) a standard spin echo Carr–Purcell–Meiboom–Gill 1D sequence (CPMG) for aqueous low molecular weight metabolites and (c) a standard diffusion-edited pulse sequence for mainly macromolecular signals such as lipoproteins. Spectra analyses (phase and baseline correction) were performed using Bruker Topspin 4.1.4. Data were expressed as metabolite percentage relative to the total investigated metabolites.

### 2.8. Statistical Analysis

Statistical analysis was performed using the nonparametric one-way repeated-measures ANOVA when three or more conditions were compared, corrected for multiple comparisons with the Bonferroni test. For each ANOVA measurement, Welch and Brown–Forsythe F-tests, which do not assume homoscedasticity and neither of which requires homogeneity of variance, were considered to correct the calculated *p*-values. The two-tailed paired Student’s *t*-test was used only when two stimulation conditions were compared. Pairwise correlations were calculated by using the Pearson r coefficient. GraphPad Prism (v.9) was used for graphical and statistical analyses. Results are shown as median values ± interquartile range (IQR). *p*-values ≤ 0.05 were considered statistically significant. In the figures, *p*-values are assigned as follows: * ≤ 0.05; ** ≤ 0.01; *** ≤ 0.001, **** ≤ 0.0001.

## 3. Results

### 3.1. Study Participants and Design

Between 15 October 2021 and 20 January 2022, we enrolled a total of 67 adult individuals [mean age ± SD: 52.7 ± 11; female/male ratio: 1.5 (40F/27M)] willing to participate in a longitudinal pilot study in which we compared a single vaccination with the quadrivalent flu Flucelvax vaccine with the antigen composition of the 2021–2022 season [A/Wisconsin/588/2019 (H1N1) pdm09 virus, A/Cambodia/e0826360/2020 (H3N2) virus; B/Washington/02/2019 virus (B/Victoria lineage); B/Phuket/3073/2013 virus (B/Yamagata lineage)] or with the COVID-19 Comirnaty vaccine (third booster dose) to the simultaneous administration of both vaccines.

Enrolled individuals were divided among the following groups (Figure 1): CoV*_only_* group consists of healthy subjects (HS) to whom only the third booster dose of COVID-19 Comirnaty vaccine was administered; Flu*_before_*CoV group comprises of HS, to whom quadrivalent flu vaccine was administered 1 month before immunization with the 3rd booster dose of COVID-19 Comirnaty vaccine; Flu*_plus_*CoV groups are composed of either HS or people with type 2 diabetes mellitus (T2D) to whom quadrivalent flu vaccine was simultaneously administered with the third booster dose of COVID-19 Comirnaty vaccine. For each group, serum samples, demographic and clinical (including vaccine-related side effects) data as well as the lifestyle characteristics of each enrolled immunized subject were longitudinally collected (Table 1) before and after 1 or 30 days from each vaccine dose as indicated in the scheme of Figure 1.

Overall, the most commonly reported local adverse events were pain and swelling at the local injection sites and this occurrence was similar in the co-administration group (21%) compared to Flu*_before_*CoV (22%) or CoV*_only_* (17%) (Table 1). The reported systemic adverse events were mainly fever, asthenia, myalgia and headache with a slightly higher occurrence in the co-administration group (fever: 24%; asthenia: 28%; myalgia: 10.5%; headache: 16%) with respect to what reported in Flu*_before_*CoV (fever: 17%; asthenia: 17%; myalgia: 0%; headache: 0%) or CoV*_only_* (fever: 12.5%; asthenia: 21%; myalgia: 4%; headache: 4%).

### 3.2. Humoral Immune Response Induced by Flu Flucelvax and COVID-19 Comirnaty Vaccines

**Figure 2:**
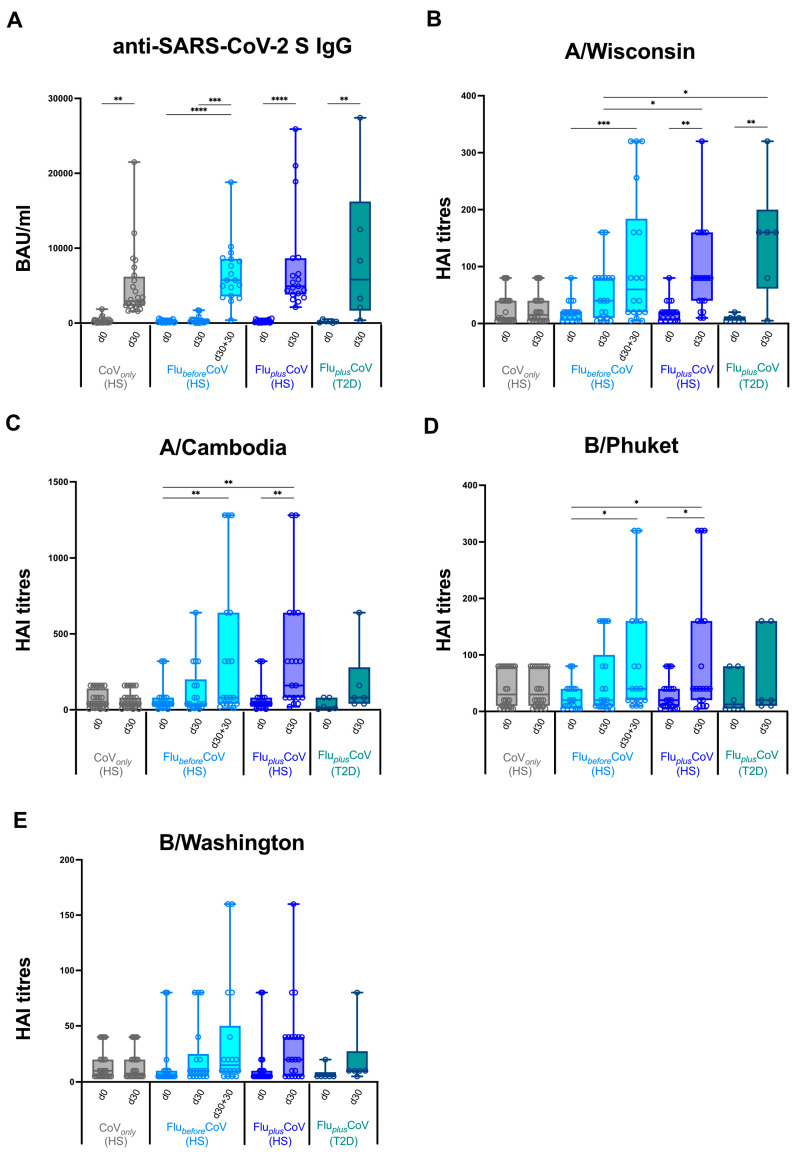
Anti-influenza and anti-COVID-19 vaccine-specific antibody production in healthy and type 2 diabetes mellitus (T2D) vaccine recipients. Levels of binding class G immunoglobulins recognizing SARS-CoV-2 trimeric spike protein (anti-SARS-CoV-2 S IgG; expressed as BAU/mL) (**A**) and specific anti-influenza (Flu) antibodies against all the vaccine antigen components included in the Flucelvax Quadrivalent flu vaccine used in the 2021–2022 season (A/Wisconsin, A/Cambodia, B/Phuket and B/Washington; expressed as hemagglutination inhibition*,* HAI, titers) (**B**–**E**) were measured in “CoV*_only_*” group (in grey), “Flu*_before_*CoV” (in light blue) as well as “Flu*_plus_*CoV” groups (in blue for healthy subjects, HS; in petrol green for T2D individuals). Serum samples were analyzed before (day 0, d0) and 30 days (d30) after the third booster dose of COVID-19 mRNA vaccine in the presence or absence of flu vaccination. Only for “Flu*_before_*CoV” subjects, sera were tested before (day 0, d0) quadrivalent flu vaccine, as well before (day 30, d30) and 30 days after the third booster dose of COVID-19 mRNA vaccine (d30 + 30). *p*-values calculated by one-way ANOVA test were assigned as follows: * ≤ 0.05; ** ≤ 0.01; *** ≤ 0.001, **** ≤ 0.0001.

Initially, we monitored antibody-mediated protection against COVID-19 or quadrivalent flu vaccines by measuring the levels of binding IgG recognizing anti-SARS-CoV-2 trimeric S protein (Figure 2A) and specific anti-flu antibodies (measured as HAI titers) against all the vaccine antigen components (here called A/Wisconsin, A/Cambodia, B/Phuket and B/Washington) (Figure 2B–E). Antibody levels were measured in serum samples longitudinally collected in CoV*_only_* and Flu*_plus_*CoV groups (both HS and T2D individuals) before (day 0, d0) and 30 days (d30) after the third booster dose of COVID-19 vaccine in the presence or absence of the co-administration of flu vaccine, as well as in the Flu*_before_*CoV group before (day 0, D0) the flu vaccine as well as before (day 30, D30) and 30 days after the third booster dose of the COVID-19 vaccine (d30 and d30 + 30, respectively) (see Figure 1).

We did not find any significant difference in the induction of anti-SARS-CoV-2 S IgG measured 30 days after COVID-19 vaccine (d30 + 30 in Flu*_before_*CoV compared to d30 in Flu*_plus_*CoV HS and T2D and in CoV*_only_*) that was strong and consistent across all the analyzed study groups (Figure 2A). Conversely, when we measured the HAI titers for flu vaccine antigen components a different picture emerged. Similar baseline levels (day 0) were observed for all the cohorts and no increase was found, as expected, at day 30 (d30) in the CoV*_only_* group that did not undergo flu vaccination (Figure 2B–E). Most importantly, if we compared day 30, very relevant differences emerged in sera from HS enrolled in Flu*_before_*CoV or Flu*_plus_*CoV groups.

When co-administration of the two vaccines was conducted, a synergistic effect was found in the induction levels of anti-flu antibodies with a statistically significant rise in the HAI titer values for all the vaccine components as compared to the values found in the single vaccination cohort. Vaccine recipients of Flu*_before_*CoV group needed 30 more days (d30 + 30), a total of two months, to reach the level of protection against flu vaccine obtained in the co-administration group already after 30 days (d30). Similar findings to that obtained for HS in the co-administration group were achieved also for the T2D vaccine recipients enrolled in Flu*_plus_*CoV (Figure 2B–E).

**Figure 3:**
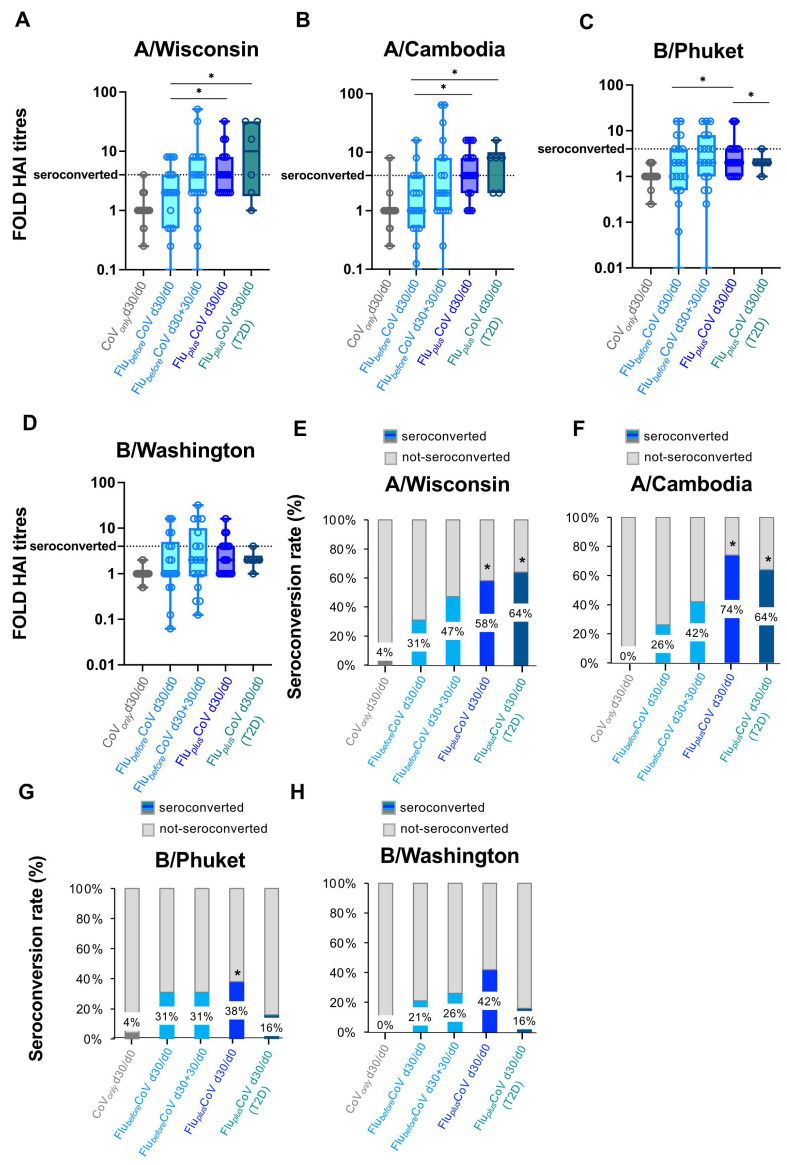
Seroconversion for influenza vaccine strains in healthy and type 2 diabetes mellitus (T2D) vaccine recipients. A comparison of seroconversion rate based on hemagglutination inhibition (HAI) titers obtained for all the vaccine antigen components included in the Flucelvax Quadrivalent influenza (flu) vaccine used in the 2021–2022 season (A/Wisconsin, A/Cambodia, B/Phuket and B/Washington) was reported for all the study groups. In particular, the seroconversion rate was calculated as a fold increase of HAI titers (**A**–**D**) and as a percentage (%) of seroconverted individuals (**E**–**H**) in “CoV*_only_*” group (in grey) and “Flu*_plus_*CoV” groups (in blue for healthy subjects, HS, and in petrol green for T2D individuals) and comparing HAI titers measured before (day 0, d0) and 30 days (d30) after the third booster dose of COVID-19 mRNA vaccine in presence or absence of flu vaccine (d30/d0). For the “Flu*_before_*CoV” group (in light blue), the seroconversion rate was calculated comparing the HAI titers obtained before (day 0, d0) and 30 or 60 days after the quadrivalent flu vaccine (d30/d0 and d30 + 30/d0, respectively). *p*-values calculated by one-way ANOVA test were assigned as follows: * ≤ 0.05.

Moreover, the statistically significant differences in the HAI titers for A/Wisconsin, A/Cambodia, B/Phuket and B/Washington observed in the co-administration group versus Flu*_before_*CoV and CoV*_only_* were also reflected in the HAI fold rises (Figure 3A–D) as well as in the seroconversion rates (Figure 3E–H). Interestingly, in Flu*_plus_*CoV groups (for both HS and T2D recipients) the seroconversion rate for flu A strains was much higher at day 30 than that calculated for the Flu*_before_*CoV group either at day 30 or 60 (d30 + 30) after the single flu vaccination (Figure 3E–H).

HAI titers and seroconversion rates obtained for flu A strains were generally higher than those observed for flu B strains (Figure 2 and Figure 3).

In line with the seroconversion data, the percentage of seroprotected individuals was also significantly greater in the co-administration group than in the single vaccination cohorts (Supplementary Figure S1A–D).

### 3.3. Innate Immune Cytokine/Chemokine Profile Induced by Flu Flucelvax and COVID-19 Comirnaty Vaccines

**Figure 4:**
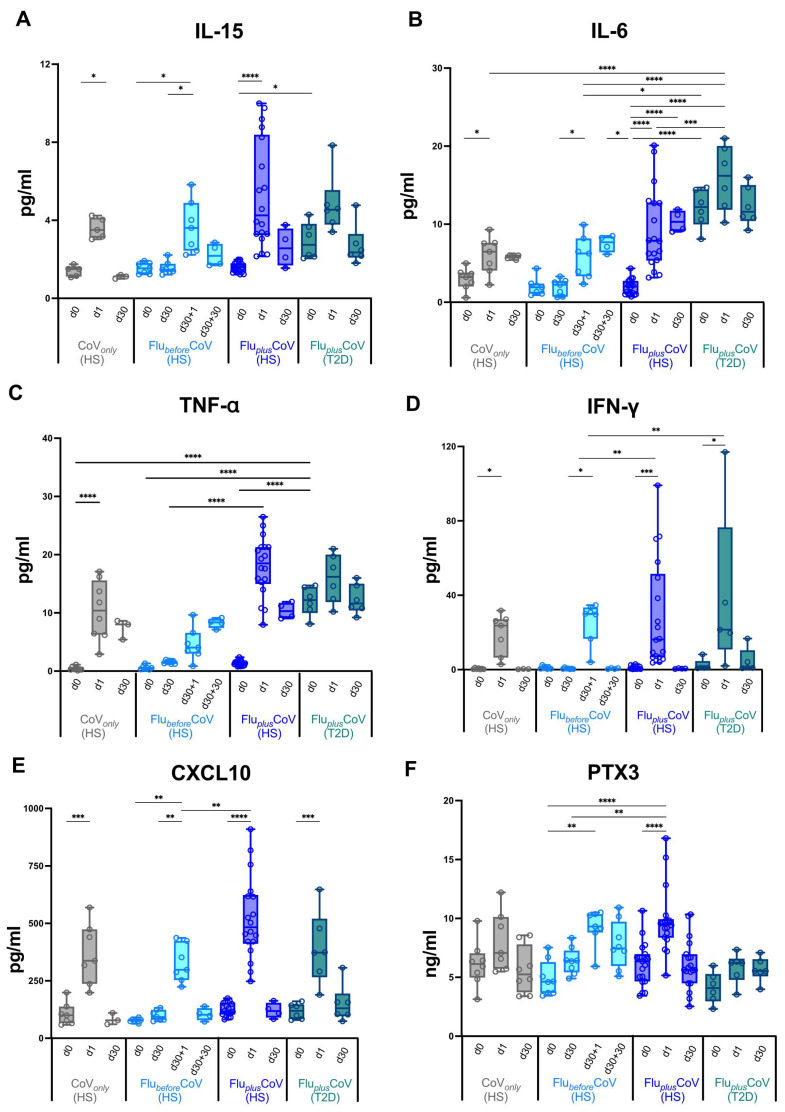
Early vaccine-induced cytokine and chemokine profile in healthy and type 2 diabetes mellitus (T2D) vaccine recipients. Level of cytokines (IL-15, IL-6, TNF-α, IFN-γ) (**A**–**D**), of the chemokine CXCL-10 (**E**) and of the acute-phase protein Pentraxin 3 (PTX3) (**F**) were measured using multiplex magnetic bead panel in serum samples longitudinally collected from the “CoV*_only_*” group (in grey) and the “Flu*_plus_*CoV” groups (in blue for healthy subjects, HS, and in petrol green for T2D individuals) immediately before (day 0, d0), as well as 1 day and 30 days (d1 and d30, respectively) after the third booster dose of COVID-19 mRNA vaccine in the presence or absence of the flu vaccine. Only for the “Flu*_before_*CoV” group (in light blue) was the analysis performed on sera stored before (day 0, d0) the quadrivalent flu vaccine, as well as before (day 30, d30) and 1 and 30 days after (d30 + 1 and d30 + 30, respectively) the third booster dose of COVID-19 mRNA vaccine. *p*-values calculated by one-way ANOVA test were assigned as follows: * ≤ 0.05; ** ≤ 0.01; *** ≤ 0.001, **** ≤ 0.0001.

This evidence and our previous data indicate a correlation between an early innate immune cytokine/chemokine serum signature and a late humoral response to vaccination [11], so we must next assess whether the simultaneous administration of the two vaccines would impact the early immunological events occurring in the innate immune compartment soon after vaccination in both the HS and T2D subjects. In the CoV*_only_* group receiving only the third booster dose of COVID-19 vaccine, we found, on 1 day (d1) post-immunization, a strong increase of the cytokines IL-15, IL-6, TNF-α and IFN-γ as well as of the chemokine CXCL10 (Figure 4A–E). The level of IL-6 also remained sustained for 30 days after the dose (Figure 4B). A comparable trend was also observed for the Flu*_before_*CoV group after COVID-19 vaccination (d30 + 1 and d30 + 30) (Figure 4A–E). Most interestingly, as seen for the antibody production, for the HS of the Flu*_plus_*CoV group, the co-administration of both flu and COVID-19 vaccines drives a significantly higher level of these serum factors (Figure 4A–E). Nonetheless, in the T2D sub-group of the Flu*_plus_*CoV cohort, while the production of IFN-γ and CXCL10 followed a similar trend to that of the HS (Figure 4C–E), the level of the inflammatory factors IL-15, IL-6 and TNF-α was already high at day 0 before vaccine co-administration and only slightly increased 1 day after, consistent with a chronic inflammation occurring in people with T2D (Figure 4A–C).

PTX3, a protein related to C-reactive protein (CRP), is an inflammatory biomarker known to be rapidly produced by several cell types upon induction with inflammatory stimuli, including adjuvants [25,26]. In a recent study, a mild increase in PTX3 circulating levels was found early after flu vaccination [27]; thus, we decided to measure PTX3 in our study groups. Induction of serum PTX3 was mainly found 1 day after the COVID-19 vaccine in Flu*_before_*CoV (d30 + 1) and increased in HS of the Flu*_plus_*CoV cohort (Figure 4F). Only a slight and not significant induction was seen instead for the T2D of the Flu*_plus_*CoV (Figure 4F).

### 3.4. Early Serum Metabolome Changes Induced by Flu Flucelvax and COVID-19 Comirnaty Vaccines

Since regulation of circulating cytokines may trigger metabolic changes at a systemic level, we wanted to verify whether the vaccine-modulated innate immune profile upon single vaccination or the co-administration of the two vaccines would impact the homeostasis of vaccine recipients at a cell, organ and organism level. To do so, we measured the circulating metabolites of both aqueous and lipidic profiles in intact serum samples of the different cohorts.

**Figure 5:**
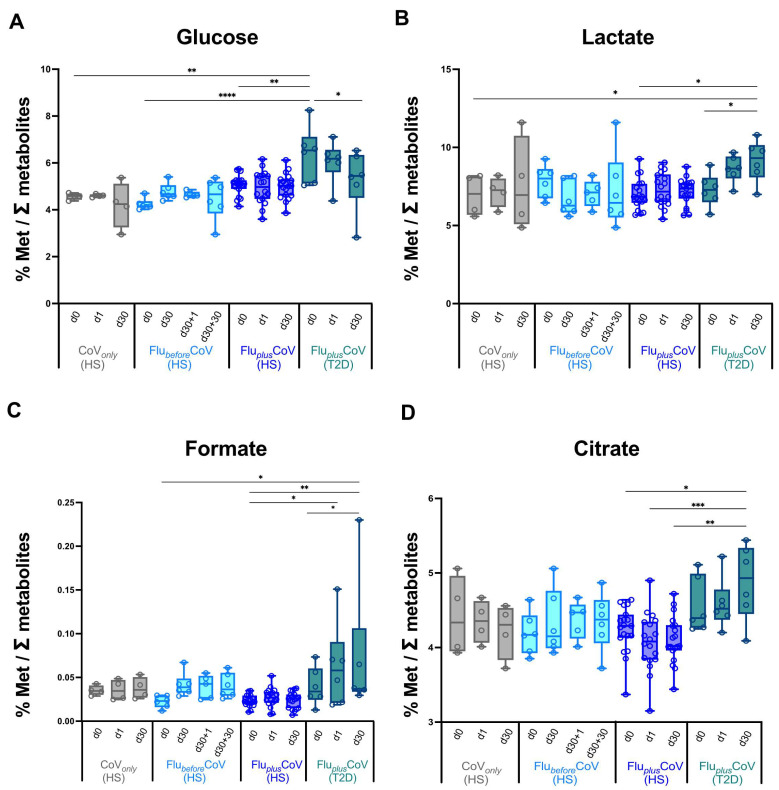
Regulation of circulating metabolites involved in glucose/pyruvate pathways in healthy and type 2 diabetes mellitus (T2D) individuals receiving influenza and COVID-19 vaccination. Changes in glucose (**A**), lactate (**B**), formate (**C**) and citrate (**D**) levels were measured in serum samples longitudinally collected in fasting condition from the “CoVonly” group (in grey) and “FluplusCoV” groups (in blue for healthy subjects, HS; in petrol green for T2D individuals) immediately before (day 0, d0), as well as 1 and 30 days (d1 and d30, respectively) after the third booster dose of the COVID-19 mRNA vaccine in presence or absence of the flu vaccine. Only for the “FlubeforeCoV” group (in light blue) was the analysis performed on sera stored before (day 0, d0) the quadrivalent flu vaccine, as well as before (day 30, d30) and 1 and 30 days after (d30 + 1 and d30 + 30, respectively) the third booster dose of COVID-19 mRNA vaccine. Values are shown as percentage (%) of the metabolite level relative to total metabolites. *p*-values calculated by one-way ANOVA test were assigned as follows: * ≤ 0.05; ** ≤ 0.01; *** ≤ 0.001, **** ≤ 0.0001.

No major difference in the modulation at early or later time points post-vaccination of the analyzed metabolites involved in glucose/pyruvate pathways, in liver and amino acid metabolism (Figure 5 and Supplementary Figure S2), as well as in lipid and fatty acid metabolism (Supplementary Figure S3) was observed indicating an overall balanced homeostatic status in vaccine recipients undergoing the single or double immunization schedules. Most interestingly, however, is that in the T2D vaccine recipients of the Flu*_plus_*CoV group, the co-administration of both the flu and COVID-19 vaccines significantly and positively impact on the glucose metabolism, reducing over time the overall glucose levels and increasing the lactate, formate and citrate, thus indicating increased oxidation of glucose digestion (consumption) in favor of the activation of downstream pyruvate pathways (Figure 5A–D).

### 3.5. Early Vaccine-Induced Immune Module Predictive of Protective Antibody Response

**Figure 6:**
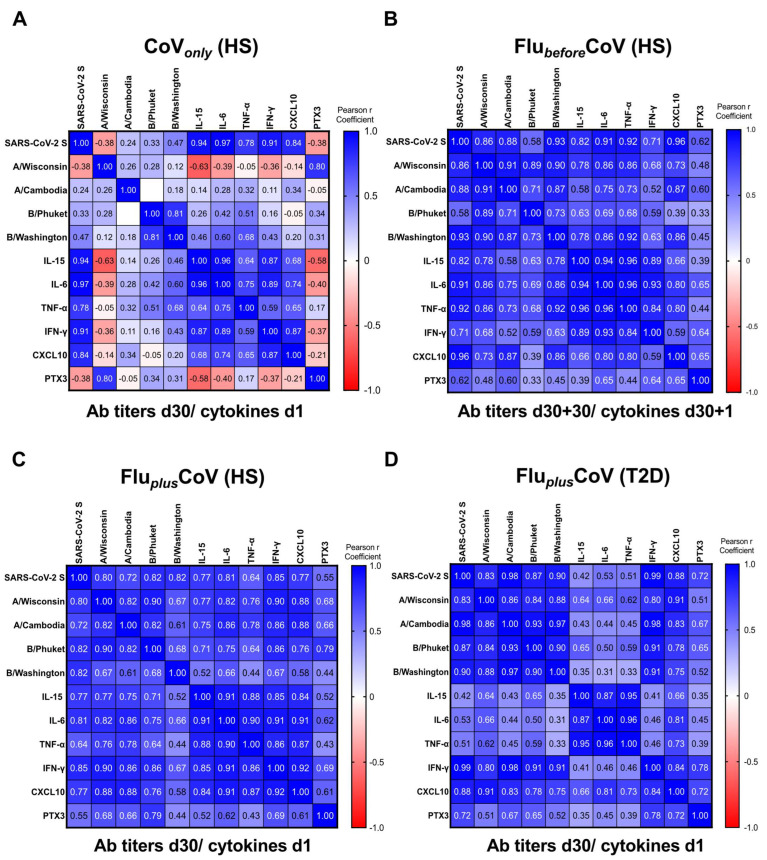
Correlation of anti-influenza and anti-COVID-19 vaccine-specific antibody production with early innate signature in healthy and type 2 diabetes mellitus (T2D) vaccine recipients. Correlations were built between levels of binding class G immunoglobulins recognizing anti-SARS-CoV-2 trimeric spike protein (anti-SARS-CoV-2 S) and specific anti-influenza (flu) antibodies against Flucelvax 2021/2022 vaccine antigen components (A/Wisconsin, A/Cambodia, B/Phuket and B/Washington) and serum level of cytokines (IL-15, IL-6, TNF-α, IFN-γ), the chemokine CXCL-10 and the acute-phase protein Pentraxin 3 (PTX3). Innate factors and vaccine antibodies were measured 1 day (d1) and 30 days (d30), respectively, after the third booster dose of COVID-19 mRNA vaccine (CoV*_only_*) (**A**), in presence of previous flu vaccination (d30 + 30 for Flu*_before_*CoV) (**B**) or co-administered with the flu vaccine (Flu*_plus_*CoV) in both healthy subjects (HS) and T2D (**C**,**D**). Correlations were obtained by deriving a Pearson r correlation coefficient (in the legend positive correlation is indicated in shades of blue, while negative correlation is indicated in shades of red).

In order to assess whether the predictive value of the early innate cytokine/chemokine signature, already exploited for COVID-19 vaccine-specific protective antibody response [11], could also be applied to vaccine co-administration, we calculated the pairwise correlations by the Pearson r coefficients comparing the early protein signature induced 1 day after vaccination with the levels of anti-SARS-CoV-2 S IgG as well as of the HAI titers for A/Wisconsin, A/Cambodia, B/Phuket and B/Washington found after 30 days from vaccine doses (Figure 6). Also, PTX3 was included in the correlation studies.

Correlation matrices were built for each study group. Results obtained from the CoV*_only_* group were in line with the previously published data [11], indicating a strong positive correlation of the serum cytokine/chemokine levels with anti-SARS-CoV-2 S humoral response (Figure 6A). For the data related to the Flu*_before_*CoV group, all the analyzed cytokines positively correlated with levels of anti-SARS-CoV-2 S antibodies 30 days after the COVID-19 vaccine (D60). Conversely, only spotted correlations were observed with HAI titers of flu strains (Figure 6B), while the Pearson r coefficients importantly increased in the HS enrolled in the Flu*_plus_*CoV group, indicating a strong correlation with the levels of all the tested antibodies (Figure 6C). Similar results were found for PTX3. Moreover, the quantity of the different inflammatory factors also positively correlated with each other (Figure 6A–D).

Regarding people with T2D enrolled in the Flu*_plus_*CoV group, we found no correlation between IL-15, IL-6 and TNF-α with COVID-19 and flu antibodies (Figure 6D), since the circulating levels of these cytokines were already high at the baseline (see Figure 4A–C). Nonetheless, the induction of the IFN-γ/CXCL10 axis was maintained in these individuals (see Figure 4D,E) as also the positive correlation of these factors with anti-vaccine humoral responses (Figure 6D).

Single linear regression curves for all the analyzed parameters referring to the Flu*_plus_*CoV groups (reporting also adjusted *p*-values) are shown in Supplementary Figures S4 and S5 (for HS and T2D vaccine recipients, respectively).

Thus, our study might be considered as a validation of the predictive value of the previously identified early immune signature, extended also to PTX3, on the magnitude of protective humoral responses to COVID-19 mRNA vaccine. Furthermore, when this vaccine was simultaneously administered with quadrivalent flu vaccination, the correlation of the early innate module was also found with vaccine-specific antibody production not only to the COVID-19 vaccine but also to the flu vaccine, differently to what was observed when flu vaccine was inoculated alone.

## 4. Discussion

In infants, children and young adolescents, vaccine co-administration is a well-established practice [28]. Co-administration of pediatric vaccines has been demonstrated to be safe and to possess an excellent immunogenicity profile [29]. For instance, the diphtheria-tetanus-acellular pertussis-hepatitis B-poliomyelitis-*Haemophilus influenzae* type b vaccine has been administered for more than 19 years and can be also used with several other infant vaccines [29]. Nonetheless, this practice is less common in adults except for travelers, for which multiple co-administered vaccines are recommended or required to be received depending on the endemic diseases at their destinations. For example, the co-vaccination for yellow fever, poliomyelitis, hepatitis B and/or A, rabies and typhoid fever is normally well tolerated [30].

An improved vaccine acceptance and uptake combined to minimal medical visits are some of the benefits associated with vaccine co-administration that lead to an increased coverage and protection against multiple diseases. In addition, combined vaccination might favor the compliance rate and, in turn, accelerate the achievement of “herd immunity” and reduce congestion in healthcare facilities and public vaccine hesitancy during mass campaigns.

With the occurrence of COVID-19 pandemic, beginning in autumn 2021, the concomitant flu and COVID-19 vaccine administration in distinct anatomic sites of adults and older adults was allowed in more than 20 countries worldwide, adhering to WHO recommendation [31]. Furthermore, the co-administration of influenza and SARS-CoV-2 vaccines into a single shot is becoming highly appealing and many clinical trials are ongoing with either co-formulated mRNA- or protein-based vaccines [32,33].

In this longitudinal pilot study, we examined the immune response to a single vaccination with either the quadrivalent flu Flucelvax vaccine (with the antigen composition of the 2021–2022 season) or with COVID-19 Comirnaty vaccine (third booster dose) when compared to the simultaneous administration of both vaccines in a group of 67 adult individuals. We found no significant increase in local or systemic adverse events in the co-administration Flu*_plus_*CoV group and comparable levels in the induction of anti-SARS-CoV-2 S IgG across all the analyzed groups. Conversely, when co-administration of the two vaccines was performed, a synergistic effect was found in the induction levels of anti-flu antibodies with a statistically significant and quicker rise in the HAI titer values for all the vaccine components as compared to that found in the single vaccination cohort. Similarly, HAI fold rises and seroconversion as well as seroprotection rates were much higher at day 30 in Flu*_plus_*CoV groups than that calculated for the Flu*_before_*CoV group either at day 30 or 60 after the single flu vaccination.

Our findings are in line with collected results from different clinical trials conducted in Europe and in the US that compared the safety and immunogenicity of the co-administration versus the mono-administration of four flu vaccines (Fluad and Flucelvax Tetra by Seqirus, and Flublok Quadrivalent or Fluzone High-Dose Quadrivalent by Sanofi Pasteur) and four COVID-19 vaccines (Vaxzevria by AstraZeneca, Nuvaxovid by Novavax, Spikevax by Moderna and Comirnaty by Pfizer/BioNTech) [34]. Whatever the vaccines or the age (< or >65) of vaccinated subjects, no safety concerns or immune interferences emerged with very similar reactogenicity profiles globally among co- and mono-administration groups. As for our results, in these studies no significant difference in anti-S IgG antibody titers was found between the co-administration groups and individuals who received COVID-19 vaccines alone [4,5,7,8,34].

A pairwise comparison conducted in a trial including different cohorts of individuals receiving concomitant vaccination with randomly assigned COVID-19 vaccines (either adenoviral- or mRNA-based) and different types of flu vaccines (cellular quadrivalent, trivalent, recombinant quadrivalent or MF59C adjuvanted) showed a preservation of the humoral IgG response to both vaccines measured at 3 weeks after immunization [5]. Nonetheless, the HAI titers to A/H1N1pdm09, B/Victoria and B/Yamagata strains were tested higher in the BNT162b2 + recombinant quadrivalent flu vaccine group than in individuals who received the flu vaccine only [5]. By comparing these data with those derived from our study, we found similar synergistic increases in HAI titers and seroprotection/seroconversion rates for A/Wisconsin, A/Cambodia and B/Phuket in the co-administration group that received the cellular quadrivalent flu vaccine. Moreover, in this study anti-flu humoral response and seroconversion obtained for flu A strains were generally higher than those observed for flu B strains.

In people with T2D, it is well known that the risk for infection and complications is increased for both flu and COVID-19 diseases [35,36]. Similarly to seasonal flu vaccination, COVID-19 vaccine administration is highly recommended in this population; thus, a simultaneous shot for both vaccines has also been practiced for them in the last few years. Even if T2D compromises innate and adaptive immune responses, young and elderly individuals with this pathology were shown to have optimal antibody response to flu vaccination, albeit with antibody levels decreased with age, as also observed in the healthy population [37]. Flu vaccination prevented hospitalization by 58% and hospitalization due to flu or pneumonia by 43% in working-age patients with T2D in (18–64 years) [38] and all-cause mortality in elderly with T2D of 65 years and above [39]. In addition, following COVID-19 vaccination, T2D people display a robust vaccine immune response and antibody seropositivity as compared to non-diabetics [36,40,41]. Diabetes affects antibody response to SARS-CoV-2 vaccination mainly among the elderly under insulin treatment and often affected also by other important co-morbidities [42]. In the T2D Flu*_plus_*CoV subgroup, we found similar serum levels of anti-SARS-CoV-2 S IgG, as well as of HAI titers, seroconversion and seroprotection rates for both vaccines as that observed in the HS of the same group, for all the four flu antigens contained in the tested vaccine. Even though this cohort of T2D subjects is very small, it is also true that is composed of a very controlled group of patients not insulin-dependent taking the same class of drugs, with no other major comorbidities and below 65 years of age (mean age ± SD: 59 ± 5). Thus, even if preliminary and in need to be validated in larger T2D cohorts, these findings could be strongly indicative of what could occur in a wider group of subjects with similar characteristics. Nonetheless, this study requires further analyses and validation before making such a general conclusion.

Therefore, co-administration of the two vaccines may have a synergistic effect in both HS and T2D people on the release of a protective humoral response to flu vaccination. This could be due to the intrinsic immunostimulating properties of the mRNA molecule that composes the COVID-19 BNT162b2 vaccine, as well as in the nanocarrier components, such as lipids and polymers, that could act as adjuvants amplifying the immune response to the flu vaccine [43]. mRNA formulation included in COVID-19 vaccines carries pseudo-uridine modification to mitigate innate immune response against exogenous mRNA. Other synthesis technologies have been applied to avoid undesirable inflammatory responses and enhance vaccine efficacy by increasing antigen production. However, these modified mRNA molecules and their delivery systems can still retain optimal adjuvanticity [44,45,46].

Nonetheless, several observational studies also reported a correlation between flu vaccine coverage in the elderly population [47,48] or hospital care employees [49] and lower COVID-19 deaths, with also a significant reduction in laboratory-confirmed SARS-CoV-2 cases and other COVID-19-related outcomes, such as hospitalization and admission to intensive care units, in flu-immunized subjects [50]. Some of these studies suggest that the underlying immunological mechanisms inducing cross-protection between flu vaccination and COVID-19 might be due to the induction of long-term metabolic and epigenetic reprogramming of innate immune cells, termed trained immunity or innate immune memory. This innate immune memory, mediated by vaccine administration, may boost immune responses following a second challenge, thus resulting in better protection against COVID-19 infection [9,51,52]. Hence, all these aspects must be taken into consideration to understand the positive effect that the flu/COVID-19 vaccine co-administration may have on the induction of enhanced anti-flu protective immunity.

In line with this view and thanks to robust evidence showing that for a correct humoral and cellular adaptive response an adequate and efficient innate immune activation is required, we recently identified an early innate immune serum signature, which included the cytokines IL-15, IL-6, TNF-α and IFN-γ and the chemokines CXCL-10, MCP-1 and MIG, induced 1 day after the second and third BNT162b2 vaccine doses, strongly correlating with a magnitude of later humoral response to vaccination [11]. In the present study, we evaluated whether the identified innate factors were regulated differently by single or combined vaccination. Similarly to previously published data [11], we found a strong increase of the cytokines IL-15, IL-6, TNF-α and IFN-γ as well as of the chemokine CXCL10 1 day post-immunization in the CoV*_only_* group receiving only the third booster dose of COVID-19 vaccine as well as in the Flu*_before_*CoV group after COVID-19 vaccination. Most interestingly, in the Flu*_plus_*CoV group the co-administration of both the flu and COVID-19 vaccines drives a significantly higher level of these serum factors. This was true especially for the healthy recipients receiving simultaneous vaccination, but also for T2D subjects in which the production of IFN-γ and CXCL10 was induced in a similar fashion to that of HS, while the inflammatory factors IL-15, IL-6 and TNF-α were already high at basal level before vaccine co-administration and only slightly increased 1 day after, consistently with cytokine dysregulation and chronic inflammation occurring in these individuals [53,54].

A significant finding is that the serum level of the PTX3 protein followed the modulation observed in the early increase of the studied cytokines and chemokines among HS. Only a slight, not significant, PTX3 induction was seen for the T2D subjects of the Flu*_plus_*CoV. PTX3 is a non-redundant component of the humoral arm of innate immunity rapidly induced as part of the acute phase response in several cell types by inflammatory cytokines, such as IL-1 and TNF, as well as by microbial moieties [26]. In a recent study, a mild increase in PTX3 circulating levels was found early after flu vaccination [27].

Studies on the response to adjuvants showed that PTX3 is induced in the site of vaccination [55] and by binding selected microbial moieties, acting as an amplifier and endogenous adjuvant of adaptive humoral responses [25,56]. For these reasons, PTX3 has been considered a bridge between the humoral arms of the innate and adaptive immune systems. Our results are in line with these data and suggest that PTX3 is part of the early innate immune response induced by the combination of the vaccines.

As already published, in subjects vaccinated with COVID-19 vaccine alone [11], the early induction of innate immune response and innate circulating factors soon after immunization is predictive of COVID-19 vaccine-specific protective antibody response induced 30 days after the third boosting dose in the CoV*_only_* group. In the Flu*_before_*CoV cohort, all the cytokines analyzed 1 day post COVID-19 vaccine positively correlated with levels of anti-SARS-CoV-2 S antibodies measured 30 days after vaccination, while only spotted correlations were observed with the HAI titers of flu strains. In the case of the simultaneous administration in the Flu*_plus_*CoV HS, results indicated a strong positive correlation of the serum cytokine/chemokine levels not only with later anti-SARS-CoV-2 S but also with anti-flu antibodies against all antigen components (and especially for flu A strains). Similar results were found for PTX3.

Regarding people with T2D enrolled in Flu*_plus_*CoV group, we found no correlation between IL-15, IL-6 and TNF-α with COVID-19 and flu antibodies, since the circulating levels of these cytokines were already high at the baseline. Nonetheless, the induction of the IFN-γ/CXCL10 axis was maintained in these individuals, as was the positive correlation of these factors with anti-vaccine humoral responses, which was low for PTX3.

Studies of systems vaccinology performed in subjects administered with different vaccine platforms mainly applied serology analysis and transcriptomic profiles of peripheral blood mononuclear cells to find immune modules for the early prediction of vaccine-specific protective immune response [15]. Together with these approaches, mass spectrometry or NMR-based metabolomics have often been used to provide insights and a deeper understanding of the immune response to vaccination [57]. Thus, we also performed a targeted metabolic analysis of the enrolled immunized subjects measuring modulation at early or later time points post-vaccination of circulating metabolites involved in glucose/pyruvate pathways, liver and amino acid metabolism as well as in lipid and fatty acid metabolism in order to comprehend the impact of vaccine co-administration at the organ, tissue and organism levels. In our study, as emerged by serum metabolomics, single vaccination or co-administration of the two vaccines would not impact the homeostasis of vaccine recipients since it left most of the analyzed metabolites of the aqueous or lipid fraction unchanged over time. In a recent paper dividing subjects undergoing COVID-19 mRNA vaccination in “high” and “low” responders based on neutralizing antibody production [58], Dagla et al. proposed a possible predictive association of the plasma level of ceramides or some amino acids, including phenylalanine, histidine and glutamine, differently regulated at 1, 22 and 90 days after vaccination with protective humoral response, in contrast with findings obtained in the serum samples collected in our study groups. Similarly, in another study, in young high responders to the flu vaccine, increased tryptophan and decreased PUFA levels were found, while fatty acid synthesis and cholesteryl esters accumulated in older high responders [59].

Very interestingly, however, we observed that in the T2D vaccine recipients of the Flu*_plus_*CoV group, the co-administration of both the flu and COVID-19 vaccines significantly reduces the overall circulating glucose level over time, and at the same time, increases lactate, formate and citrate, thus indicating increased oxidation of glucose digestion (consumption) in favor of the activation of downstream pyruvate pathways. This regulation of the glucose metabolism did not necessarily correlate with antibody levels; however, it could positively impact on T2D pathology. This could be due to the connection and close interaction between immune and endocrine systems. During pathogen infection, immune system activation may modify the responsiveness of the peripheral organs to endocrine signals, resulting in altered levels of blood hormones such as insulin, which promotes the ability of the body to fight infection [60]. Similarly, a boost of immune response upon vaccine administration may regulate glucose metabolism in T2D patients.

Altogether, our results suggest that the COVID-19/flu vaccine co-administration schedule firstly amplifies in a synergistic way the magnitude of protective humoral response and seroconversion to flu vaccination as compared to the single immunization in both healthy and T2D subjects, and secondly, increases the oxidation of glucose digestion reducing the overall circulating glucose level in T2D vaccinees. Furthermore, our findings confirm the applicability of an innate serum protein signature, composed of IL-15, IL-6, TNF-α, IFN-γ, CXCL10 and extended to PTX3, that might act as an early indicator of vaccine-induced protection not only in COVID-19 vaccination but also in the simultaneous immunization of COVID-19 and flu vaccines, even to T2D subjects.

This study, although with several limitations, and even if conducted in a small group of individuals thus requiring further investigation and validation in larger cohorts, provides insights into the safety and immunogenicity of the simultaneous administration of COVID-19 and flu vaccines and could be useful in the future to build up personalized vaccination strategies, especially in frail categories, including those with chronic disorders such as diabetes.

## Figures and Tables

**Table 1:** Demographic, clinical and lifestyle characteristics of enrolled immunized subjects.

										FOR T2D ONLY	
	Code	Sex	Age	Comorbidity	Therapy	Lifestyle (Sedentary or Sporty)	Administration of Flu Vaccine in Previous Vaccination Seasons	Confirmed SARS-CoV-2 Infection	Post-COVID-19 III DOSE Vaccination Side Effects	GLYCATED HEMOGLOBIN (AT DAY 0)	LDL (mg/dL) (AT DAY 0)
**Flu*_before_*CoV group (HS, *n* = 18)**	Co C1	F	59	mild hypercholesterolemia	n.r.	sporty	yes	NO	n.r.		
	Co C2	M	49	n.r.	n.r.	not sedentary, physically inactive	yes	NO	n.r.		
	Co C3	F	42	hypothyroidism	eutirox	not sedentary, physically inactive	yes	NO	pain at the inoculum site, fever, asthenia		
	Co C5	F	30	n.r.	n.r.	sporty	yes	NO	pain at the inoculum site, fever, asthenia		
	Co_C6	F	65	n.r.	n.r.	not sedentary, physically inactive	yes	NO	n.r.		
	Co_C9	M	58	n.r.	n.r.	sedentary, physically inactive	yes	NO	n.r.		
	Co_C10	M	65	n.r.	n.r.	sedentary, physically inactive	yes	NO	n.r.		
	Co_C16	F	52	n.r.	n.r.	not sedentary, physically inactive	yes	NO	n.r.		
	Co_C17	F	49	n.r.	n.r.	not sedentary, physically inactive	yes	NO	n.r.		
	CO_C_204	M	48	n.r.	n.r.	not sedentary, physically inactive	yes	NO	n.r.		
	CO_C_205	F	29	n.r.	n.r.	sporty	yes	NO	n.r.		
	CO_C_206	F	47	n.r.	n.r.	not sedentary, physically inactive	yes	NO	n.r.		
	CO_C_210	F	51	n.r.	n.r.	not sedentary, physically inactive	yes	NO	n.r.		
	CO_C_211	M	31	n.r.	n.r.	sporty	no	NO	pain at the inoculum site, fever, asthenia		
	CO_C_212	F	32	n.r.	n.r.	sporty	yes	NO	n.r.		
	CO_C_213	F	38	n.r.	n.r.	not sedentary, physically inactive	no	NO	n.r.		
	CO_C_214	M	48	n.r.	n.r.	sedentary, physically inactive	yes	NO	n.r.		
	CO_C_215	M	28	n.r.	n.r.	not sedentary, physically inactive	no	NO	swelling at the inoculum site, asthenia		
**Flu*_plus_*CoV group (HS, *n* = 19)**	CoFlu C1	M	60	n.r.	n.r.	sedentary, physically inactive	yes	NO	n.r.		
	CoFlu C2	F	63	hypercholesterolemia, hypertension	losartan, rosuvastatina	sedentary, physically inactive	yes	yes	asthenia		
	CoFlu C3	F	59	hypothyroidism	eutirox	sporty	yes	NO	n.r.		
	CoFlu C4	M	65	n.r.	n.r.	sedentary, physically inactive	yes	NO	pain at the inoculum site		
	CoFlu C7	M	64	n.r.	n.r.	sedentary, physically inactive	yes	NO	fever		
	CoFlu C8	M	60	n.r.	n.r.	sporty	yes	NO	myalgia, fever		
	CoFlu C9	F	58	n.r.	n.r.	sedentary, physically inactive	yes	NO	none		
	CoFlu C10	F	65	n.r.	n.r.	sedentary, physically inactive	yes	NO	athralgia		
	CoFlu C11	F	33	n.r.	n.r.	sporty	yes	NO	pain at the inoculum site		
	CoFlu C12	M	65	n.r.	n.r.	not sedentary, physically inactive	yes	NO	n.r.		
	CoFlu C13	M	62	n.r.	n.r.	not sedentary, physically inactive	yes	NO	n.r.		
	CoFlu C14	F	51	n.r.	n.r.	not sedentary, physically inactive	yes	NO	asthenia, myalgia, headache, fever		
	CoFlu C15	M	56	hypertension	norvasc	sedentary, physically inactive	yes	NO	n.r.		
	CoFlu C16	F	53	n.r.	n.r.	sporty	yes	NO	n.r.		
	CoFlu C17	F	58	n.r.	n.r.	not sedentary, physically inactive	yes	NO	n.r.		
	CoFlu C18	F	59	asthma	relvar	not sedentary, physically inactive	yes	NO	asthenia, headache, pain at the inoculum site		
	CoFlu C19	M	63			not sedentary, physically inactive	yes	NO	asthenia		
	CoFlu C20	F	46	hypothyroidism	eutirox	not sedentary, physically inactive	yes	NO	headache, fever		
	CoFlu C21	F	56	mild tachycardia	cardicor	not sedentary, physically inactive	yes	NO	none		
**Flu*_plus_*CoV group (T2D, *n* = 6)**	CoFlu D1	M	55	hypertension	metformin, blopress	sedentary, physically inactive	yes	NO	fever, asthenia, headache	5,5%	98
	CoFlu D2	F	63	hypertension, hypothyroidism	metformin, cacit, janumet, triveram, eutirox	sedentary, physically inactive	yes	NO	asthenia	6%	97
	CoFlu D3	M	52	hypercholesterolemia	metformin, simvastin	not sedentary, physically inactive	yes	yes	n.r.	6,1%	114,8
	CoFlu D4	M	64	retinopathy, neuropathy, vasculopathy	metformin, SGLT2-inhibitor	not sedentary, physically inactive	no	yes	n.r.	5,7%	67
	CoFlu D201	F	57	hypercholesterolemia, hypertension	pioglitazone, irbesartan	sedentary, physically inactive	yes	NO	swelling at the inoculation site, fever	6,7%	98
	CoFlu D202	M	64	hypercholesterolemia	metformin, statins	not sedentary, physically inactive	yes	NO	asthenia	6%	98
**CoV*_only_* group (HS, *n* = 24)**	Co C4	F	42	n.r.	n.r.	not sedentary, physically inactive	yes	NO	swelling at the inoculum site, asthenia		
	Co_C7	M	59	n.r.	n.r.	sedentary, physically inactive	NO	NO	n.r.		
	Co_C8	M	60	n.r.	n.r.	sedentary, physically inactive	NO	NO	n.r.		
	Co_C11	F	48	n.r.	n.r.	sedentary, physically inactive	NO	NO	n.r.		
	Co_C12	F	31	n.r.	n.r.		NO	NO	n.r.		
	Co_C13	F	42	n.r.	n.r.	not sedentary, physically inactive	yes	NO	swelling at the inoculum site, fever, asthenia		
	Co_C14	F	36	n.r.	n.r.	sporty	NO	NO	n.r.		
	Co_C15	F	41	n.r.	n.r.	not sedentary, physically inactive	NO	NO	n.r.		
	CO_C_101	F	52	n.r.	n.r.	not sedentary, physically inactive	NO	NO	n.r.		
	CO_C_102	F	50	n.r.	n.r.	not sedentary, physically inactive	NO	NO	n.r.		
	CO_C_103	F	22	n.r.	n.r.	sporty	NO	NO	swelling at the inoculum site, fever, asthenia		
	CO_C_104	M	60	n.r.	n.r.	not sedentary, physically inactive	yes	NO	n.r.		
	CO_C_105	M	45	n.r.	n.r.	not sedentary, physically inactive	NO	NO	n.r.		
	CO_C_106	F	52	n.r.	n.r.	not sedentary, physically inactive	NO	NO	n.r.		
	CO_C_107	F	50	n.r.	n.r.	sporty	NO	NO	n.r.		
	CO_C_108	F	22	n.r.	n.r.	sporty	NO	NO	n.r.		
	CO_C_109	F	42	n.r.	n.r.	sporty	NO	NO	n.r.		
	CO_C_201	F	44	n.r.	n.r.	sedentary, physically inactive	NO	NO	n.r.		
	CO_C_202	M	47	n.r.	n.r.	not sedentary, physically inactive	NO	NO	n.r.		
	CO_C_203	M	41	n.r.	n.r.	not sedentary, physically inactive	NO	NO	n.r.		
	CO_C_207	M	31	n.r.	n.r.	sporty	NO	NO	asthenia, myalgia, headache, fever		
	CO_C_208	F	41	n.r.	n.r.	sporty	NO	NO	asthenia		
	CO_C_209	F	31	n.r.	n.r.	sporty	NO	NO	n.r.		
	CO_C_216	M	52	n.r.	n.r.	not sedentary, physically inactive	NO	yes	n.r.		

HS: healthy subjects; T2D: subjects with type 2 diabetes mellitus; M: male; F: female; n.r.: not reported.

## Data Availability

Data generated during the study will be made available after a direct request to the corresponding authors.

## Supplementary Materials

The following supporting information can be downloaded at: https://www.mdpi.com/article/10.3390/vaccines12091050/s1.
