# Supplementary material for: A Serum Multi-Parametric Analysis Identifies an Early Innate Immune Signature Associated to Increased Vaccine-Specific Antibody Production and Seroconversion in Simultaneous COVID-19 mRNA and Cell-Based Quadrivalent Influenza Vaccination
Source: Vaccines (Basel). 2024 Sep 13;12(9):1050. doi: 10.3390/vaccines12091050 (PMC11436141; doi:10.3390/vaccines12091050)
Supplement: Supplementary file 1 [file vaccines-12-01050-s001.zip › vaccines-3138482-supplementary.pptx]

## Slide 1
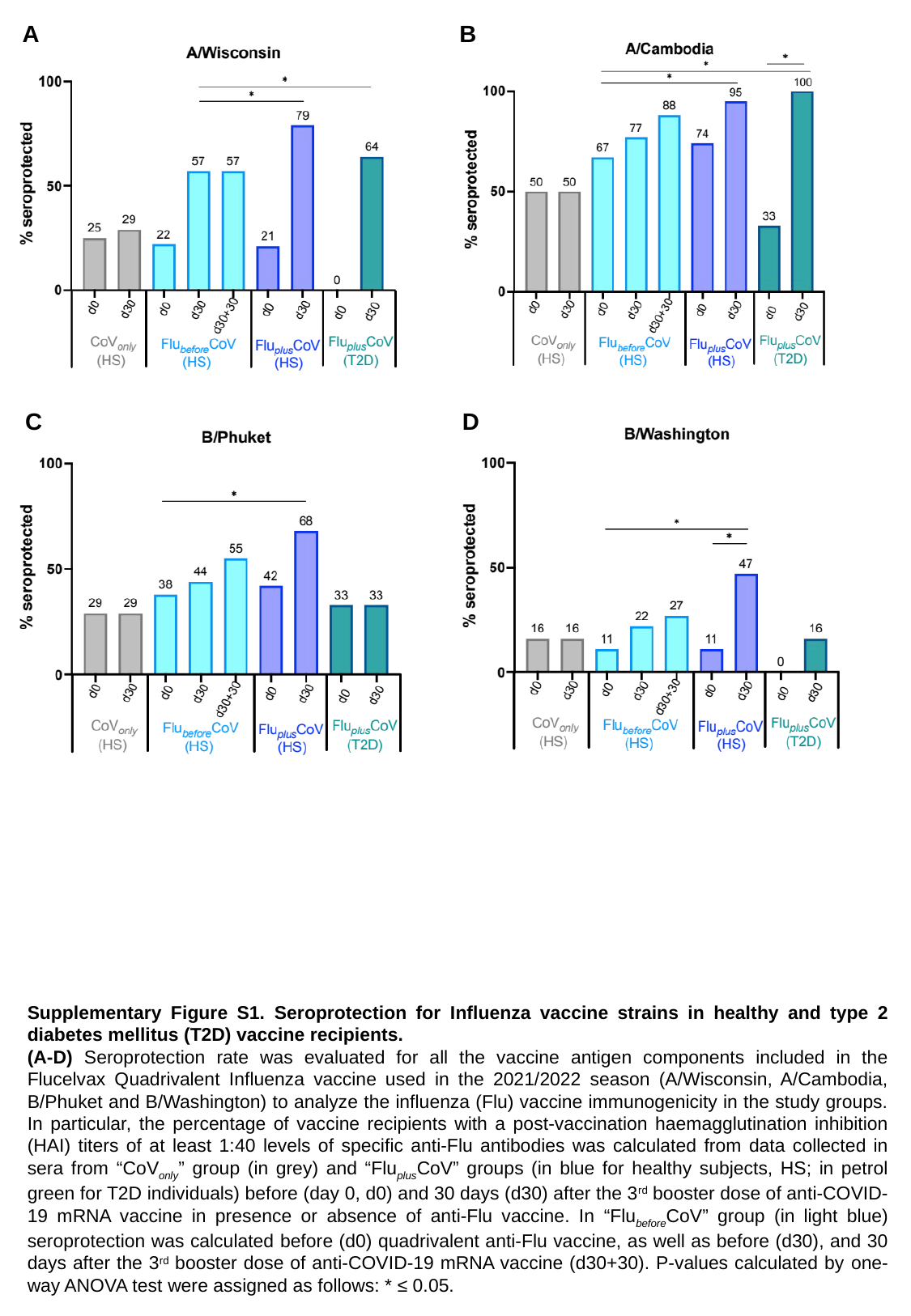

A
B
C
D
Supplementary Figure S1. Seroprotection for Influenza vaccine strains in healthy and type 2 diabetes mellitus (T2D) vaccine recipients.
(A-D) Seroprotection rate was evaluated for all the vaccine antigen components included in the Flucelvax Quadrivalent Influenza vaccine used in the 2021/2022 season (A/Wisconsin, A/Cambodia, B/Phuket and B/Washington) to analyze the influenza (Flu) vaccine immunogenicity in the study groups. In particular, the percentage of vaccine recipients with a post-vaccination haemagglutination inhibition (HAI) titers of at least 1:40 levels of specific anti-Flu antibodies was calculated from data collected in sera from “CoVonly” group (in grey) and “FluplusCoV” groups (in blue for healthy subjects, HS; in petrol green for T2D individuals) before (day 0, d0) and 30 days (d30) after the 3rd booster dose of anti-COVID-19 mRNA vaccine in presence or absence of anti-Flu vaccine. In “FlubeforeCoV” group (in light blue) seroprotection was calculated before (d0) quadrivalent anti-Flu vaccine, as well as before (d30), and 30 days after the 3rd booster dose of anti-COVID-19 mRNA vaccine (d30+30). P-values calculated by one-way ANOVA test were assigned as follows: * ≤ 0.05.

## Slide 2
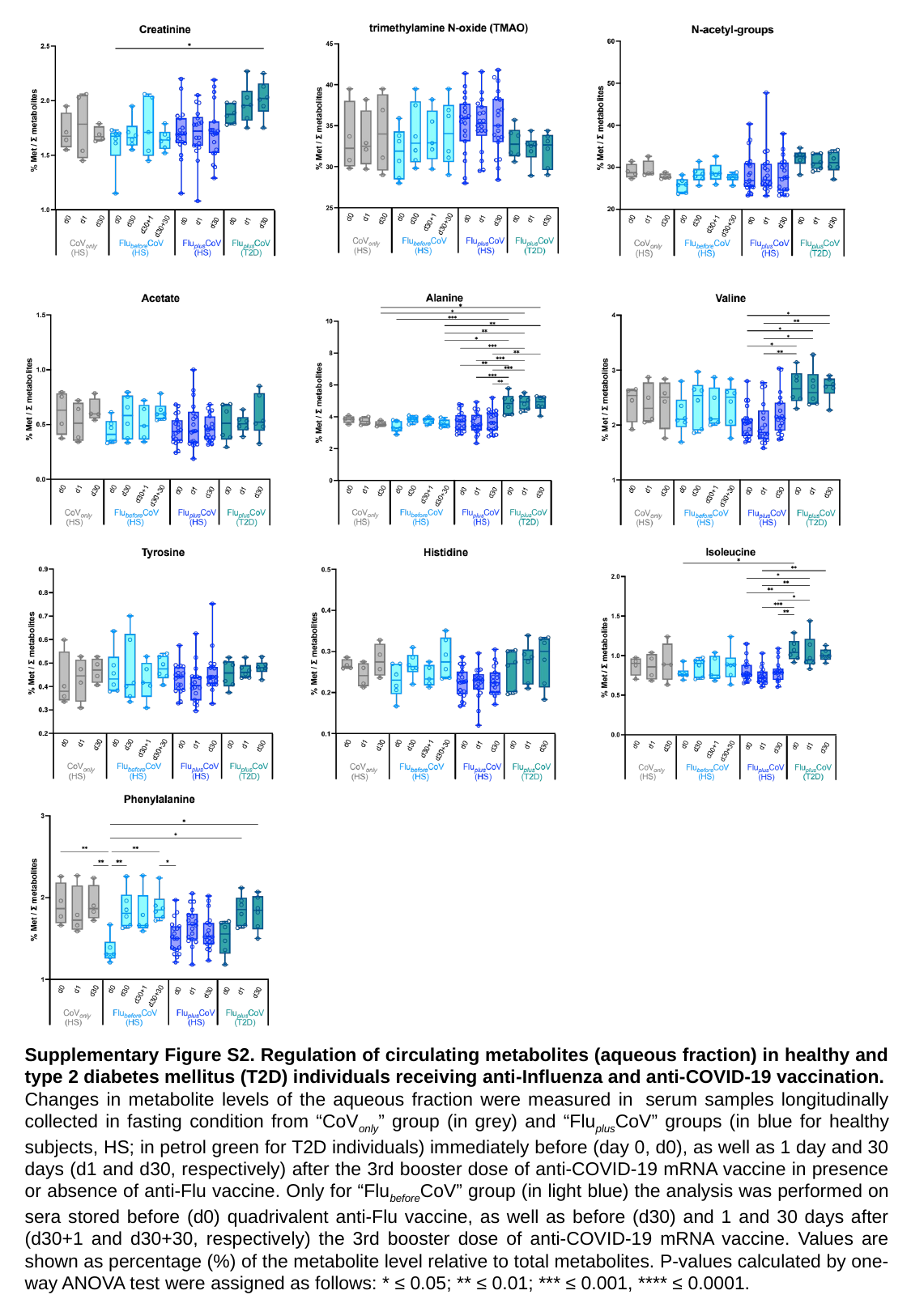

Supplementary Figure S2. Regulation of circulating metabolites (aqueous fraction) in healthy and type 2 diabetes mellitus (T2D) individuals receiving anti-Influenza and anti-COVID-19 vaccination.
Changes in metabolite levels of the aqueous fraction were measured in  serum samples longitudinally collected in fasting condition from “CoVonly” group (in grey) and “FluplusCoV” groups (in blue for healthy subjects, HS; in petrol green for T2D individuals) immediately before (day 0, d0), as well as 1 day and 30 days (d1 and d30, respectively) after the 3rd booster dose of anti-COVID-19 mRNA vaccine in presence or absence of anti-Flu vaccine. Only for “FlubeforeCoV” group (in light blue) the analysis was performed on sera stored before (d0) quadrivalent anti-Flu vaccine, as well as before (d30) and 1 and 30 days after (d30+1 and d30+30, respectively) the 3rd booster dose of anti-COVID-19 mRNA vaccine. Values are shown as percentage (%) of the metabolite level relative to total metabolites. P-values calculated by one-way ANOVA test were assigned as follows: * ≤ 0.05; ** ≤ 0.01; *** ≤ 0.001, **** ≤ 0.0001.

## Slide 3
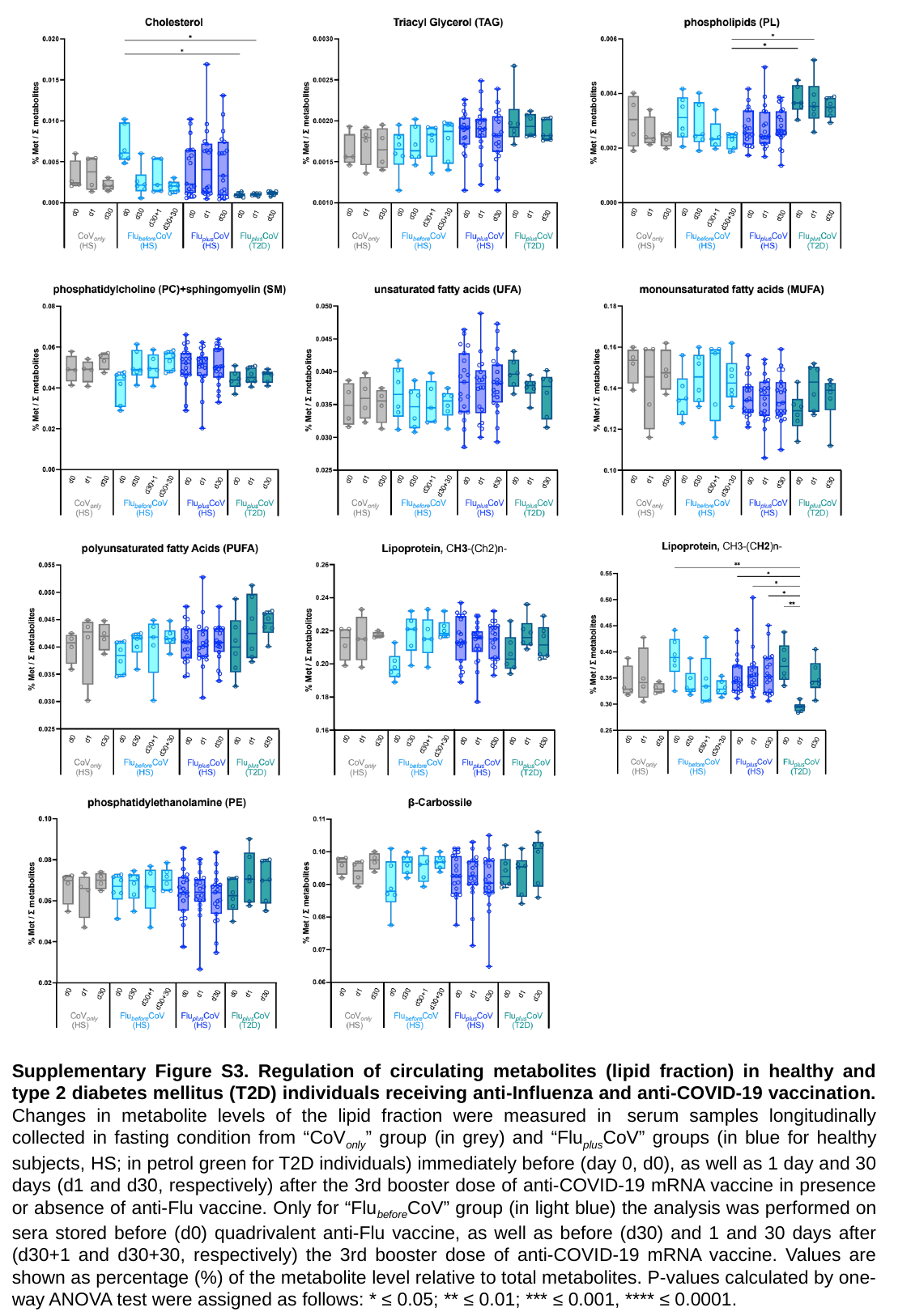

Supplementary Figure S3. Regulation of circulating metabolites (lipid fraction) in healthy and type 2 diabetes mellitus (T2D) individuals receiving anti-Influenza and anti-COVID-19 vaccination.
Changes in metabolite levels of the lipid fraction were measured in  serum samples longitudinally collected in fasting condition from “CoVonly” group (in grey) and “FluplusCoV” groups (in blue for healthy subjects, HS; in petrol green for T2D individuals) immediately before (day 0, d0), as well as 1 day and 30 days (d1 and d30, respectively) after the 3rd booster dose of anti-COVID-19 mRNA vaccine in presence or absence of anti-Flu vaccine. Only for “FlubeforeCoV” group (in light blue) the analysis was performed on sera stored before (d0) quadrivalent anti-Flu vaccine, as well as before (d30) and 1 and 30 days after (d30+1 and d30+30, respectively) the 3rd booster dose of anti-COVID-19 mRNA vaccine. Values are shown as percentage (%) of the metabolite level relative to total metabolites. P-values calculated by one-way ANOVA test were assigned as follows: * ≤ 0.05; ** ≤ 0.01; *** ≤ 0.001, **** ≤ 0.0001.

## Slide 4
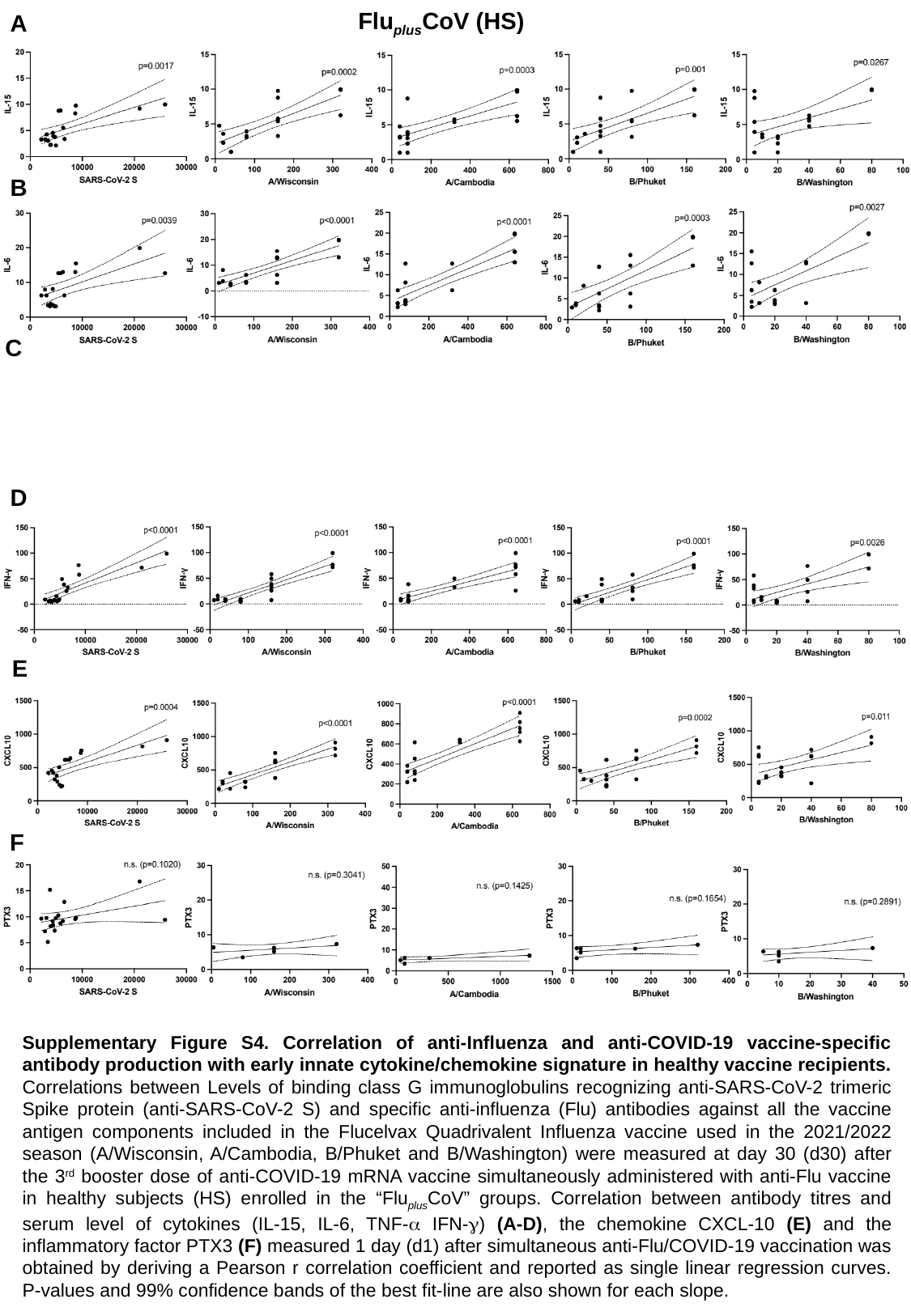

FluplusCoV (HS)
A
B
C
D
E
F
Supplementary Figure S4. Correlation of anti-Influenza and anti-COVID-19 vaccine-specific antibody production with early innate cytokine/chemokine signature in healthy vaccine recipients.
Correlations between Levels of binding class G immunoglobulins recognizing anti-SARS-CoV-2 trimeric Spike protein (anti-SARS-CoV-2 S) and specific anti-influenza (Flu) antibodies against all the vaccine antigen components included in the Flucelvax Quadrivalent Influenza vaccine used in the 2021/2022 season (A/Wisconsin, A/Cambodia, B/Phuket and B/Washington) were measured at day 30 (d30) after the 3rd booster dose of anti-COVID-19 mRNA vaccine simultaneously administered with anti-Flu vaccine in healthy subjects (HS) enrolled in the “FluplusCoV” groups. Correlation between antibody titres and serum level of cytokines (IL-15, IL-6, TNF-a IFN-γ) (A-D), the chemokine CXCL-10 (E) and the inflammatory factor PTX3 (F) measured 1 day (d1) after simultaneous anti-Flu/COVID-19 vaccination was obtained by deriving a Pearson r correlation coefficient and reported as single linear regression curves. P-values and 99% confidence bands of the best fit-line are also shown for each slope.

## Slide 5
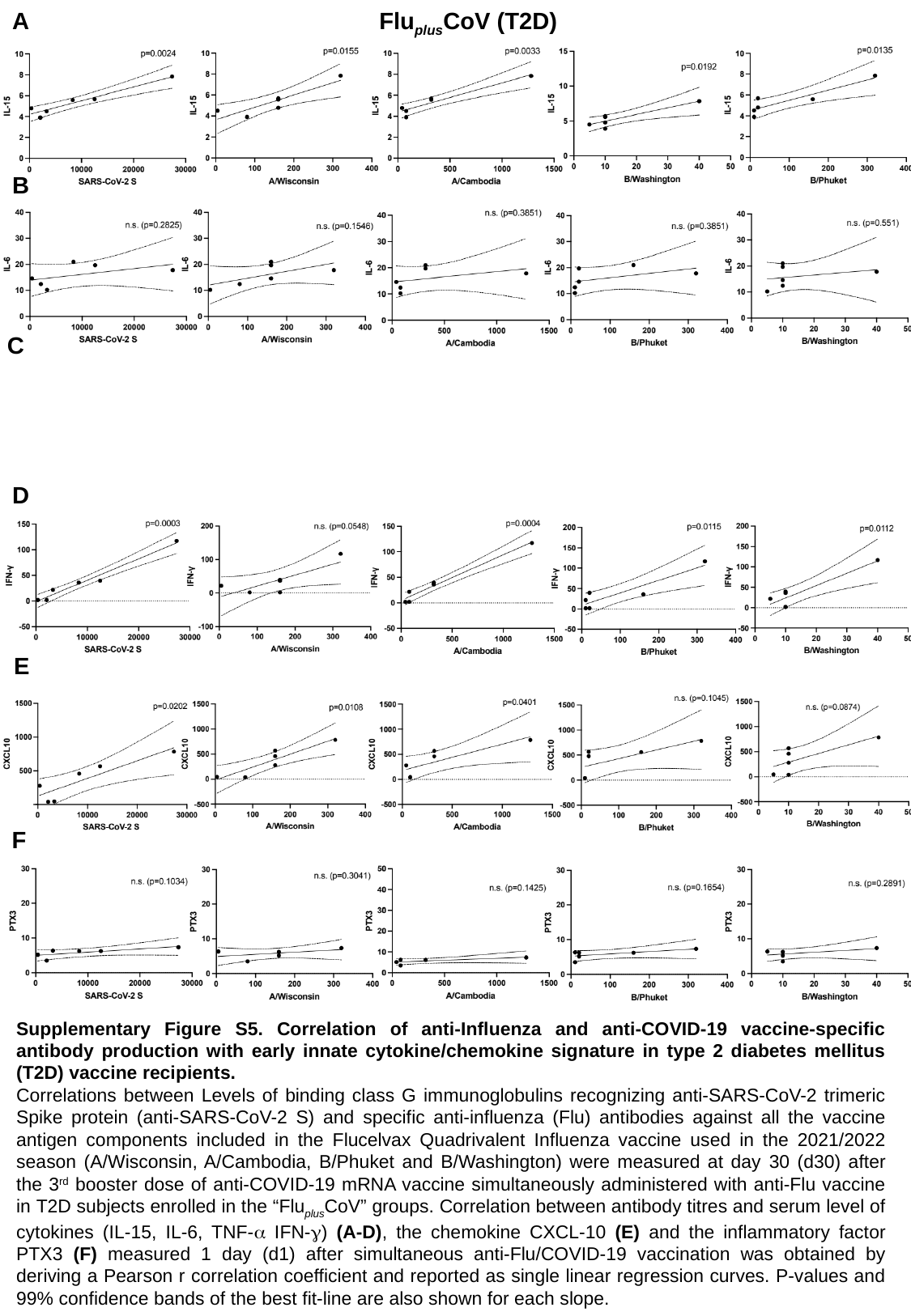

A
FluplusCoV (T2D)
B
C
D
E
F
Supplementary Figure S5. Correlation of anti-Influenza and anti-COVID-19 vaccine-specific antibody production with early innate cytokine/chemokine signature in type 2 diabetes mellitus (T2D) vaccine recipients.
Correlations between Levels of binding class G immunoglobulins recognizing anti-SARS-CoV-2 trimeric Spike protein (anti-SARS-CoV-2 S) and specific anti-influenza (Flu) antibodies against all the vaccine antigen components included in the Flucelvax Quadrivalent Influenza vaccine used in the 2021/2022 season (A/Wisconsin, A/Cambodia, B/Phuket and B/Washington) were measured at day 30 (d30) after the 3rd booster dose of anti-COVID-19 mRNA vaccine simultaneously administered with anti-Flu vaccine in T2D subjects enrolled in the “FluplusCoV” groups. Correlation between antibody titres and serum level of cytokines (IL-15, IL-6, TNF-a IFN-γ) (A-D), the chemokine CXCL-10 (E) and the inflammatory factor PTX3 (F) measured 1 day (d1) after simultaneous anti-Flu/COVID-19 vaccination was obtained by deriving a Pearson r correlation coefficient and reported as single linear regression curves. P-values and 99% confidence bands of the best fit-line are also shown for each slope.
